# Learning what to expect (in visual perception)

**DOI:** 10.3389/fnhum.2013.00668

**Published:** 2013-10-24

**Authors:** Peggy Seriès, Aaron R. Seitz

**Affiliations:** ^1^Department of Informatics, University of EdinburghEdinburgh, UK; ^2^Department of Psychology, University of California at RiversideRiverside, CA, USA

**Keywords:** expectations, Bayesian priors, statistical learning, perceptual learning, probabilistic inference

## Abstract

Expectations are known to greatly affect our experience of the world. A growing theory in computational neuroscience is that perception can be successfully described using Bayesian inference models and that the brain is “Bayes-optimal” under some constraints. In this context, expectations are particularly interesting, because they can be viewed as prior beliefs in the statistical inference process. A number of questions remain unsolved, however, for example: How fast do priors change over time? Are there limits in the complexity of the priors that can be learned? How do an individual’s priors compare to the true scene statistics? Can we unlearn priors that are thought to correspond to natural scene statistics? Where and what are the neural substrate of priors? Focusing on the perception of visual motion, we here review recent studies from our laboratories and others addressing these issues. We discuss how these data on motion perception fit within the broader literature on perceptual Bayesian priors, perceptual expectations, and statistical and perceptual learning and review the possible neural basis of priors.

## INTRODUCTION

Our perceptions are strongly shaped by our expectations. In ambiguous situations, knowledge of the world guides our interpretation of the sensory information and helps us recognize objects and people quickly and accurately, although sometimes leading to illusions (Bar, 2004; Summerfield and Egner, 2009). Expectations are formed at various levels of sensory processing and appear to be continuously updated. Indeed, statistical and perceptual learning studies show that the visual system continuously extracts and learns the statistical regularities of the environment, and can do so automatically and without awareness. This knowledge is then used to modulate information acquisition and interpretation (e.g., Perruchet and Pacton, 2006; Fiser et al., 2010).

In parallel to the experimental study of expectations, a growing body of theoretical work suggests that visual perception is akin to Bayesian Inference (e.g., Knill and Pouget, 2004; Colombo and Seriès, 2012; Fiser et al., 2010; Friston, 2012). This idea, which is thought to find its origins in Helmholtz’s notion of “unconscious inference” (see, e.g., Westheimer, 2008), provides an ideal theoretical framework for the study of expectations. Bayesian models propose that, at each moment in time, the visual system uses implicit knowledge of the environment to infer properties of visual objects from ambiguous sensory inputs. This process is thought to be automatic and unconscious. In mathematical terms, to say that a system performs Bayesian inference is to say that it updates the probability *P*(*H*∣*D*) that a hypothesis *H* is true given some data *D* by executing Bayes’ rule:

$$\;\;P\left(H|D\right)=\frac{P\left(D|H\right)\,\;\;P\left(H\right)}{P\left(D\right)}$$

In visual perception, the hypothesis *H* could correspond to the presence of a visual target (detection task) or a value of a given stimulus (estimation task), while *D* describes the visual input. *P*(*D*∣*H*) measures how compatible the data is with the hypothesis and is called the “likelihood.” The “prior” *P*(*H*) corresponds to one’s prior expectations about the probability of the hypothesis, and serves to interpret the data in situations of uncertainty. The more uncertain the data, the more the prior influences the interpretation. Optimal priors should reflect previous experience with the sensory world. Together, the likelihood *P*(*D*∣*H*) and the prior *P*(*H*) make up the “generative model.”

The study of expectations, of statistical and perceptual learning, and the so-called “Bayesian Brain hypothesis” have developed somewhat independently. However, it is very fruitful to consider how these fields can inform each other and potentially be unified. A number of questions remain unsolved, in particular: How fast do prior expectations change over time? Are there limits in the complexity of the priors that can be learned? How do priors compare to the true stimulus statistics in individuals? Can we unlearn priors that are thought to correspond to natural scene statistics? We here review work from our lab and others investigating these questions. Section “Expectations and Visual Priors” begins with an effort to define and classify perceptual priors and their influence on perception. Focusing on visual perception (and even more particularly, motion perception), we review how perceptual priors can be measured in individuals and the relation between internal priors and “true” environment distributions. The next section focuses on learning of new priors. We then address whether there is a limitation to the complexity of the priors that can be learned. The following section asks whether long-term priors are fixed or whether they can be updated. We then review the potential neural substrate of perceptual priors. We conclude with outstanding issues and promising research directions.

## EXPECTATIONS AND VISUAL PRIORS

### CONTEXTUAL AND STRUCTURAL EXPECTATIONS

While visual expectations likely originate from diverse mechanisms, we propose that they fall into two broad categories, “contextual” and “structural,” based upon the extent to which they generalize across environmental circumstance. Briefly, “*contextual*” expectations have impact in isolated spatial or temporal situations, whereas “*structural*” expectations impact all perceptions of the stimulus features to which they relate.

**FIGURE 1:**
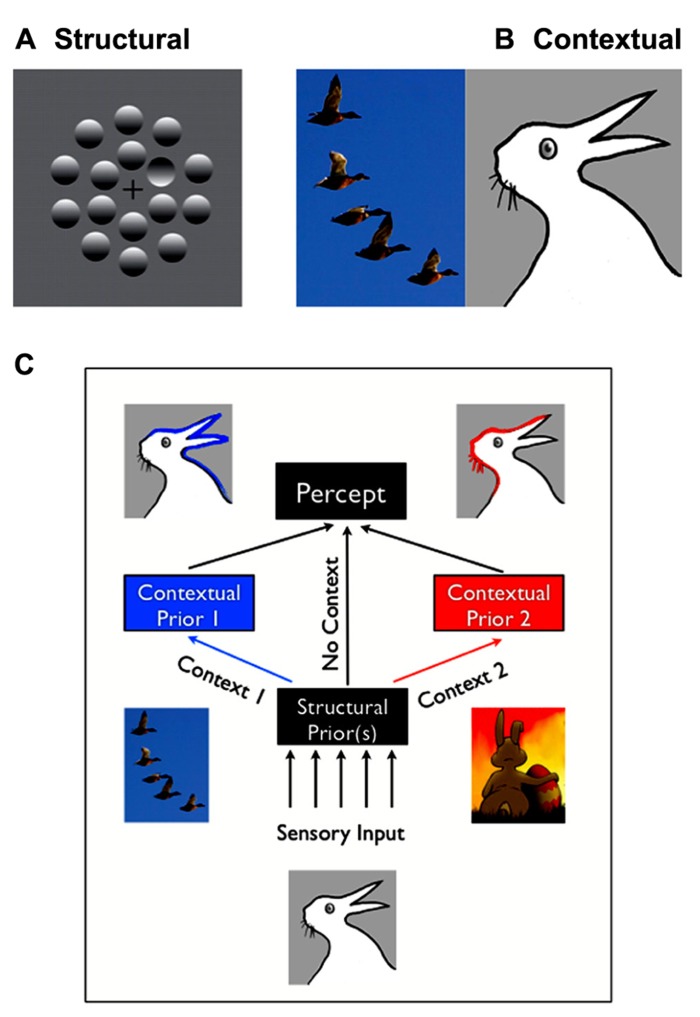
**Structural vs. contextual expectations. (A)** Example of a structural expectation: the “light-from-above” prior. Are those shapes bumps or dimples? Perceiving one dimple in the middle of bumps is consistent with assuming that light comes from the top of the image. Turning the page upside down would lead to the opposite percept (seeing a bump in a middle of dimples). **(B)** Example of a contextual expectation. What do you see in the drawing on the right: a rabbit or duck? This ambiguous and bistable percept can be influenced by the spatial context in which it is placed, for, e.g., having just seen a flock of ducks would make one more likely the perceive a duck. **(C)** Structural expectations act as “default” expectations, but can be superseded by contextual expectations.

Structural expectations are the “default” expectations that human observers use based on implicit learning of the statistics of the natural environment. These expectations usually reflect long-term learning over the lifetime, or may be innate. For example, in **Figure 1A**, you’ll likely see one (concave) “dimple” among (convex) “bumps” due to structural expectation that light comes from above, and thus the top of bumps should be lit while the tops of dimples should be in shadow. A characteristic of structural expectations is that they apply broadly to how observers see the world, including novel images.

Contextual expectations, on the other hand, can be manipulated rapidly, explicitly or implicitly, through instructions (e.g., Sterzer et al., 2008; “the same stimulus will be repeated”), sensory cues (e.g., Posner, 1980; an arrow indicating that a stimulus will appear on the right), or by the spatial, temporal, or stimulus context in which a stimulus is shown (Chun and Jiang, 1998; Haijiang et al., 2006). For example, the presence of the flock of ducks in **Figure 1B** (left) will increase the probability that you’ll perceive a duck in the bistable image on the right, rather than a rabbit. Conversely, you’d be more likely to interpret it as being a rabbit on Easter day than in October (Brugger and Brugger, 1993). Other interesting examples of contextual expectations can be found in the domain of figure-ground segregation. Convexity, for example, is known to be a powerful configural cue: convex shapes are more likely to be perceived as foreground objects (a structural expectation). However, this bias also varies with the number and color of the other convex and concave regions present in the visual scene (Peterson and Salvagio, 2008). Such examples demonstrate that the spatial or temporal context can create expectations that greatly impact perceptual interpretation, but are typically short-lived and unlikely to impact all future experiences with similar objects.

The distinction between contextual and structural expectations is not specific to vision but applies to all modalities and a broad range of cognitive processes. In speech perception, for example, expectation for certain words depends on the topic of the conversation, and on a shorter time-scale, on the immediately preceding words in the same sentence (contextual expectations). However, it is also related to the overall frequency distribution of words in the language (structural expectations; see, e.g., Norris and McQueen, 2008).

In practice, there are cases where the classification of expectations into discrete categories appears to be ambiguous. Based on our findings and others, we here propose that several factors enter into play. First, structural expectations can be modulated or masked by contextual expectations (**Figure 1C**) – but these modulations will remain specific to the context. For example, participants might learn that in a given environment, light does not come from above but from a slightly different source location (Kerrigan and Adams, 2013). However, their estimation would remain unchanged in a different context. Moreover, if the context is broad or ambiguous enough, contextual expectations might appear to function like structural expectations. For example, a few trials of experience may lead to an expectation that a target may appear at a particular location in a particular context (e.g., taking into account that people drive on the left, when crossing the street in the UK). However, many thousands of such trials may lead to a structural expectation that will appear to generalize to different contexts (e.g., being confused about where to look when being back in the continent, see also Outstanding Questions).

In this review, we focus on structural expectations (and their potential contextual modulation) and use the term “prior” when they have been studied or described with the Bayesian framework in mind.

### HOW DO EXPECTATIONS IMPACT PERCEPTION?

Expectations generally can have two different types of effects on perception. First, expectations modulate perceptual performance; for example, by increasing participants’ speed and accuracy at detecting stimuli that are presented at an expected location (Sekuler and Ball, 1977; Posner, 1980; Downing, 1988), or by improving the recognition of objects that are expected within the context of a visual scene (Bar, 2004). Second, expectations can alter the subjective appearance of visual stimuli, i.e., the content of perception. These changes in perceptual appearance are strongest when the available sensory inputs are ambiguous or when there are multiple competing interpretations for the sensory input (Adams et al., 2004; Haijiang et al., 2006; Sterzer et al., 2008). **Figure 1B** illustrates this effect for contextual expectations. Visual illusions have long been used to yield insight into the structural prior assumptions that the visual system makes in interpreting the world. The expectation that light shines from above our heads, a.k.a. the “light-from-above prior,” illustrated in **Figure 1A**, is often cited in this context. Although various aspects of this prior have been debated (Mamassian and Goutcher, 2001; Morgenstern et al., 2011), it is commonly thought to determine shape interpretation and visual search performances for shaded objects. Similarly, it has been shown that human observers have a priori expectations for symmetry (e.g., Knill, 2007), smoothness or “good continuation” in space and time (e.g., Schwartz et al., 2007; Geisler and Perry, 2009), that cardinal orientations are more frequent than other orientations (e.g., Girshick et al., 2011), that objects are convex and backgrounds homogenously colored (Goldreich and Peterson, 2012) and that other people’s gaze is directly toward them (Mareschal et al., 2013). Such studies have commonly formalized their findings using a Bayesian framework, which leads to precise, quantitative predictions regarding the relationships between the sensory variables.

### A WELL-STUDIED EXAMPLE: THE SLOW-SPEED PRIOR

The so-called “slow-speed prior,” i.e., the prior belief that visual objects are static or move slowly, is one of the best studied structural expectations and will thus be a major topic in this review. This prior was first introduced as an elegant hypothesis that could provide a unified explanation for a number of visual motion illusions or biases (Weiss et al., 2002). Weiss et al. (2002) formulated a Bayesian model of visual motion perception that assumed that local image measurements are noisy and that slower motions are *a priori* more likely than faster ones (a Gaussian prior centered on 0°/s speed), a reasonable assumption in a world where most objects are static or moving slowly. They showed that this model, while leading to improved performance on average for naturalistic stimuli (compared to a model without a prior), could also account qualitatively for a wide range of biases and illusions previously observed in psychophysics: the “aperture problem” (Hildreth, 1984), the “Thomson effect,” i.e., the influence of contrast on perceived grating speed (Stone and Thompson, 1992), the rhombus illusion (Weiss et al., 2002), the influence of contrast on perceived plaid direction (Stone et al., 1990), on perceived line direction (Lorenceau and Shiffrar, 1992), on the perceived direction of Type 1 vs. type 2 plaids (Yo and Wilson, 1992), influence of relative orientation (Burke and Wenderoth, 1993), and relative speed on type 2 plaids (Bowns, 1996). They thus suggested that motion illusions may not be “the result of sloppy computation by various components in the visual system, but rather a result of a coherent computational strategy that is optimal under reasonable assumptions,” and that “visual illusions [could be viewed] as optimal percepts.” An advantage of studying the slow-speed prior is that it provides an explanation for a wide range of phenomena in motion perception and exemplifies the characteristics that we use to define structural expectations. Interestingly, similar priors have been postulated in other sensory systems. A slow-speed prior has been proposed to act in tactile perception, where it explains a variety of spatiotemporal illusions, including the cutaneous rabbit illusion, in which successive taps delivered to a couple of skin positions are perceived as a sequence of taps traveling from one position to the other, although no stimulation was applied between the two actual stimulus locations (see, e.g., Goldreich and Tong, 2013). Similar models could explain sensory saltation and length contraction illusions occurring in vision and audition (see, e.g., Geldard, 1976; Bremer et al., 1977).

### ESTIMATING PRIORS IN INDIVIDUALS

While the use of such priors in Bayesian frameworks provides a parsimonious explanation of many phenomena at a qualitative level, a key question is whether they can also inform us quantitatively on performance and internal beliefs at the level of individuals. When investigating the slow-speed prior, Weiss et al. (2002) had assumed a standard (Gaussian) shape for the prior and showed that it could qualitatively explain observers’ group performances. More recently, a number of laboratories have developed approaches to infer individuals’ priors from their behavioral responses. The general methodology is to assume that participants’ data can be accounted for by a Bayesian observer, which is specified by choosing a noise model for the sensory estimation process, a noise model for the motor response, the form of the prior and a loss function (e.g., Chalk et al., 2010; Acerbi et al., 2012). The full model is then used to fit perceptual performances, choosing the best parameters commonly by maximizing the likelihood of the data under the model (see e.g., Adams et al., 2010; Chalk et al., 2010; Gekas et al., 2013). Bayesian model comparison is often used to assess which model of a family provides the best description of the data (where different models correspond to different assumptions about the components, e.g., the form of the prior or loss function). The most common method for specifying the prior is to assume a particular parametric form (e.g., a Gaussian). The difficulty is in choosing the form of the parametric distribution, without overly constraining it, where, on the other hand, too many parameters for the prior distributions might lead to over-fitting. A few studies have tried to avoid strong parametric forms (Stocker and Simoncelli, 2006; Acerbi et al., 2012; Zhang et al., 2013). Stocker and Simoncelli (2006), for example, developed a method for estimating the prior based on measurements of both perceptual biases and variability, without constraining it to be Gaussian nor even unimodal (but assuming instead that the log of the prior is linear over the range of velocities corresponding to the width of the likelihood function). They show that the recovered priors have significantly heavier tails than a Gaussian: they fall instead with speed as a power law, with significant variability between participants. Moreover, they find individual differences in the shape of the speed prior that can be used to explain individual differences in performance.

Following this study, a number of laboratories (including ours) are now trying to link individual differences in priors’ shapes with individual differences in performance on perceptual tasks (different from those used to infer the prior). Collecting such data is recognized as a very promising way to assess the validity of the Bayesian approach (Maloney and Mamassian, 2009). However, this also raises the question: why would prior distributions differ across individuals in the first place? What are the processes that give rise to these priors? We address these questions in the following sections.

### DO STRUCTURAL PRIORS MATCH ENVIRONMENT DISTRIBUTIONS?

A natural question is whether observers’ measured prior distributions match the environment statistics, a condition for optimality (Ma, 2012). This is difficult to answer for the slow-speed prior. Indeed, it is difficult to measure the statistical distribution of retinal image velocities, because these depend not only on the statistics of natural images but also on the relative effects of body, head, and eye movements (Stocker and Simoncelli, 2006).

However, Girshick et al. (2011) successfully explored this issue in the context of visual orientation biases, applying a method similar to that of Stocker and Simoncelli (2006). They studied the performances of participants comparing different orientations, and found that participants were strongly biased toward the cardinal axes when the stimuli were uncertain. They further measured the distribution of local orientations in a collection of photographs and found that it was strongly non-uniform, with a dominance of cardinal directions. They found that the recovered priors matched the measured environmental distribution.

Another strong indication that humans use priors that are matched with the statistics of the environment comes from the recent work of Zhang et al. (2013). These authors reasoned that the slow-speed prior should hold only for foveal or parafoveal vision. In peripheral vision, when we are in motion or tracking an object, the optic flow is predominantly expanding. If prior distributions are learned from experience, the velocity prior in the visual periphery should thus correspond to faster motions, biased toward centrifugal directions. They tested this idea experimentally by measuring perceived direction of motion for peripheral gratings. They found that stationary objects in the visual periphery are indeed often perceived as moving centrifugally, while objects moving as fast as 7°/s toward the fovea are perceived as stationary. They showed that these illusions are well-explained by a Bayesian observer model that has a strong centrifugal prior in peripheral vision.

These data show that at least some structural priors approximate natural stimulus statistics. It is thus reasonable to conjecture that structural priors form as a mechanism to optimize one’s sensory processes in reflection of the environment. One question that is unclear, though, is the time-scale with which priors should change. Is the learning of priors a continual process that occurs through the lifespan of an individual? If this is the case then we should be capable of learning new priors (see Can New Priors Be Learned? and What Level of Complexity of a Prior Can Be Learned?) and update existing priors [see Can Long-Term Structural Priors Be Updated (Or Over-Ridden)?].

## CAN NEW PRIORS BE LEARNED?

Substantial research shows that contextual priors can be quickly learned. In fact, much of the research that gives evidence for contextual priors does so by inducing them experimentally. Compelling examples can be found in the perception of bistable or ambiguous displays (e.g., Adams et al., 2004; Haijiang et al., 2006; Sterzer et al., 2008). For example, the appearance of a bistable image such as the moving direction of a rotating Necker cube can be influenced by external cues when those cues have been previously associated with a particular direction for the cube (Haijiang et al., 2006).

However, there is little work concerning learning or updating of structural priors in visual perception. Theories of statistical learning suggest that, with extensive experience, mechanisms that lead to contextual priors, such as contextual cueing (Chun and Jiang, 1998) can develop into new structural priors and engage similar mechanisms as those that lead to the formation of language and visual Gestalt grouping laws (Fiser and Aslin, 2001, 2002). One way to approach structural prior learning is thus to investigate (contextual) learning paradigms that impact on the implicit use of structural expectations: can one learn through exposure to use a new statistical model for basic features of the environment, such as depth or motion cues?

Knill (2007) explored how participants’ learned expectations about stimulus shape alter their interpretation of depth. When participants are asked to judge the planar orientation of randomly shaped ellipses, they initially exhibit expectations for regularly shaped objects, and are thus biased to perceive elliptical stimuli as circles presented at an oblique angle. However, Knill (2007) found that prolonged exposure to a stimulus distribution that included a large number of randomly shaped ellipses reduced this bias. After training, participants’ learned expectations influenced how they combined different visual cues in their estimates of stimulus slant: participants gave progressively less weight to stimulus shape, and more weight to stereoscopic cues.

**FIGURE 2:**
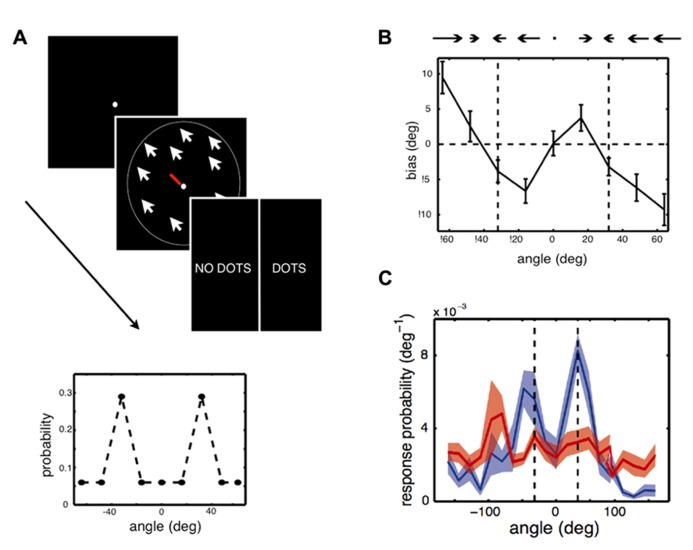
**Experiment and main results of Chalk et al. (2010). (A)** Stimulus and task used in the experiment. In each trial, participants were asked to give an estimate of the direction of motion of a cloud of coherently moving dots by moving the central bar (estimation task), then indicate whether they had perceived a stimulus or not, by clicking on “dots” or “no dots” (detection task). Some trials had very low contrast stimuli or no stimuli at all. Feedback was only given relative to the detection task. Inset: Two directions of motion, -32° and 32°, were presented in more trials than other directions. The question was whether participants would implicitly learn about this underlying stimulus distribution and how this would influence their performances. **(B)** Participants quickly exhibited attractive estimation biases: they tended to perceive motion direction as being more similar to the most frequent directions, -32° and 32° (vertical dashed lines), than they really were. **(C)** On trials when there was no stimulus but participants reported seeing a stimulus (blue line), they tended to report directions close to -32° and 32° (vertical dashed lines). When they correctly reported that there was no stimulus (red line), their estimation was more uniform.

Chalk et al. (2010) asked whether expectations formed through statistical learning could also modulate the perception of simple visual features, such as a motion direction, in a situation where there is only one available visual cue. This was examined in a design where some motion directions were more likely to appear than others (**Figure 2**). In each trial, participants were presented with either a low contrast random dot kinematogram, moving coherently in one direction, or a blank screen. Participants performed a dual task in which they were required to first report the direction of motion (estimation) and then report whether the stimulus was present (detection). Chalk et al. (2010) used a bimodal distribution of motion directions such that two directions, 64° apart from each other, were more frequently presented than the others. The hypothesis was that participants would automatically learn which directions were most likely to be presented and that these learned expectations would bias their perception of motion direction.

Chalk et al. (2010) found that after a few minutes of task performance, participants perceived stimuli to be moving in directions that were more similar to the most frequently presented directions than they actually were (attractive estimation bias). Furthermore, on trials where no stimulus was presented, but where participants reported seeing a stimulus, they were strongly biased to report motion in these two directions (a form of hallucination). No such effect was observed when participants did not report seeing a stimulus. This learning was implicit: when asked about the stimulus distribution after the experiment, most participants indicated no conscious knowledge that some directions had been presented more frequently than others. Modeling of participants’ behavior showed that their estimation biases could not be well-explained by a simple response bias or by more complex response strategies. On the other hand, the results were well-accounted for by a model which assumed that a learned prior of the stimulus statistics, corresponding to participants’ distributions of perceived motion directions in the absence of a stimulus, was combined with sensory evidence in a probabilistically optimal way. The model also provided correct predictions for participants’ behavior when no stimulus was presented. Overall, these results show that stimulus statistics are rapidly learned and can powerfully influence perception of simple visual features, both in the form of perceptual biases and hallucinations.While this research is suggestive that new structural priors can be formed, research is still lacking regarding how long-lived these effects are and the extent to which they generalize across contexts, especially to novel conditions (see also Outstanding Questions). Perceptual learning studies, however, suggest that such effects can persist over time. For example, in Seitz et al. (2005), participants were trained to notice and later report white letters presented in a series of darker letters, where unbeknownst to them, coherent motion stimuli were presented at a sub-threshold contrast level, with a specific direction of motion always paired with the target letters. This task-irrelevant perceptual learning training (Seitz and Watanabe, 2009) induced direction-specific visual hallucinations and improvements in discriminating that motion direction, in a manner similar to Chalk et al. (2010). Furthermore, participants improved in their critical flicker fusion thresholds (Seitz et al., 2006) and these improvements lasted over 6-months. While these results have not fully been characterized within a Bayesian model, they are consistent with the broad impact that structural priors can have on the visual system.

## WHAT LEVEL OF COMPLEXITY OF A PRIOR CAN BE LEARNED?

An interesting question is to understand the precision that can be achieved in learning prior distributions. For example, in the study of Chalk et al. (2010), although the prior that individual participants learned was usually sensible, it was always only an approximation of the true stimulus distribution, with high variability between individuals. A common opinion is that the brain can only achieve sub-optimal inference (Fiser et al., 2010; Beck et al., 2012) and that there are strong limits on the types of statistical regularities that sensory systems can automatically detect. However, which aspects of stimuli statistics can be learned, how it depends on the underlying complexity and what is the impact of the approximations made in the inference is unclear (Turk-Browne et al., 2008; Turk-Browne and Scholl, 2009; Berniker et al., 2010; Fiser et al., 2010; Acerbi et al., 2012; Gekas et al., 2013).

Berniker et al. (2010) recently investigated whether participants can learn the variance of the prior, in addition to the mean. They addressed this question using a visuo-motor “coin catching” experiment. They found that the mean and variance of a time-varying Gaussian prior could be learned quickly and accurately, but at different rates, with learning of the prior variance requiring more trials than learning of the mean.

In a similar spirit, Gekas et al. (2013) explored whether participants could learn two different distributions simultaneously (see also Kerrigan and Adams, 2013). They did this by modifying the experimental paradigm used in Chalk et al. (2010) to include interleaved moving dot displays of two different colors, either red or green, with different motion direction distributions. The aim of the experiment was to assess whether participants could learn the frequency distribution of motion directions of each color and whether knowledge about the statistical properties of the two distributions transferred between conditions.

When one distribution was uniform and the other bimodal (experiment 1), participants quickly developed expectations for the most frequently presented directions over all trials, irrespective of the color of the dots. They exhibited similar estimation biases toward those directions for both the uniform and bimodal color conditions. Consistent with this, on trials where no stimulus was presented but participants reported seeing a stimulus, they were strongly biased to make estimates in the most frequently presented directions regardless of the color reported. Participants’ estimation behavior was described successfully by a non-optimal Bayesian inference strategy, which combined sensory evidence with a unique learned prior of the combined stimulus statistics, applied to both color conditions in a probabilistic way.

However, when both distributions were similarly structured and chosen such that the combined distribution was uniform (experiment 2), there was evidence for the formation of two distinct priors. Participants’ estimation performances on trials where no stimulus was presented but where they reported seeing a stimulus were significantly different depending on the color they reported. Moreover, participants increasingly perceived the most frequently presented directions of the color condition they reported as the sessions progressed. For a number of participants, estimation performances were best accounted for by a model that assumed a distinct prior for each color condition. Moreover, the prior distributions for each color condition were compatible with participants’ behavior in trials where no stimulus was presented.

These results suggest that it is possible to learn the joint statistics of the stimuli but only under specific conditions. Even so, there was a tendency for participants to learn a complex combination of the two distributions and use it non-specifically in the different conditions. Interestingly, complexity does not seem to be a limiting factor per se as the distributions of experiment 2 were more complex than that of experiment 1. More relevant is probably the degree of overlap between the two stimulus distributions. Further experiments are now needed to understand what other factors impact learning in such situations. It is possible in particular that, for the visual system, plasticity, the formation, and/or use of new priors are computationally costly and that this cost needs to be balanced against possible gains in performance or expected rewards. It might also be the case that higher-level priors also enter into play. Participants might have a preference for simple explanations of their sensory input. For example, participants may have a prior expectation that similar objects might follow similar distributions. Similarly, when forming an internal model of the environment, they might have a preference for assigning stimuli to as small a set of categories (here corresponding to different motion distributions) as possible, only creating new perceptual categories when the stimulus statistics are radically different (Anderson, 1991; Sanborn et al., 2010).

## CAN LONG-TERM STRUCTURAL PRIORS BE UPDATED (OR OVER-RIDDEN)?

While we have provided evidence that human observers exhibit structural expectations that are thought to correspond to the long-term statistics of natural scenes, one may ask: are these expectations hard-wired, or fixed after long-term exposure, or are they constantly updating through experience?

This question was first addressed in the context of the light-from-above prior. Hershberger (1970) showed that chickens reared in an environment illuminated from below did not differ from controls in their interpretation of shadows and depth. They thus suggested that the prior that light comes from above is innate. Adams et al. (2004) revisited this question in humans. In their experiment, they first asked participants to make convex–concave judgments of bump-dimple stimuli at different orientations (as in **Figure 1B**), and measured the light-from-above prior based on their responses. During a training phase, they then added new shape information via haptic (active touch) feedback, that disambiguated object shape but conflicted with the participants’ initial interpretation, by corresponding to a light source shifted by 30° compared to the participants baseline prior. When participants were finally tested again on visual only stimuli, their light direction prior had shifted significantly in the direction of the information provided during training. Adams et al. (2010) thus concluded that, unlike in chickens, the “light-from-above” prior could be updated in humans. Adams et al. (2010) subsequently found that such recalibration could also be obtained using visual feedback alone.

Sotiropoulos et al. (2011a) revisited this question in the context of the slow-speed prior (Weiss et al., 2002; Stocker and Simoncelli, 2006). Although never directly tested, the speed prior is commonly thought to develop over the course of our lifetime, in a world where static or slowly moving objects are more frequent than fast objects. Sotiropoulos et al. (2011a) investigated whether expectations about the speed of visual stimuli could be changed implicitly solely through exposure (i.e., without introducing feedback or a conflict between modalities) and if so, whether this could result in a disappearance or reversal of the classically reported direction biases.

**FIGURE 3:**
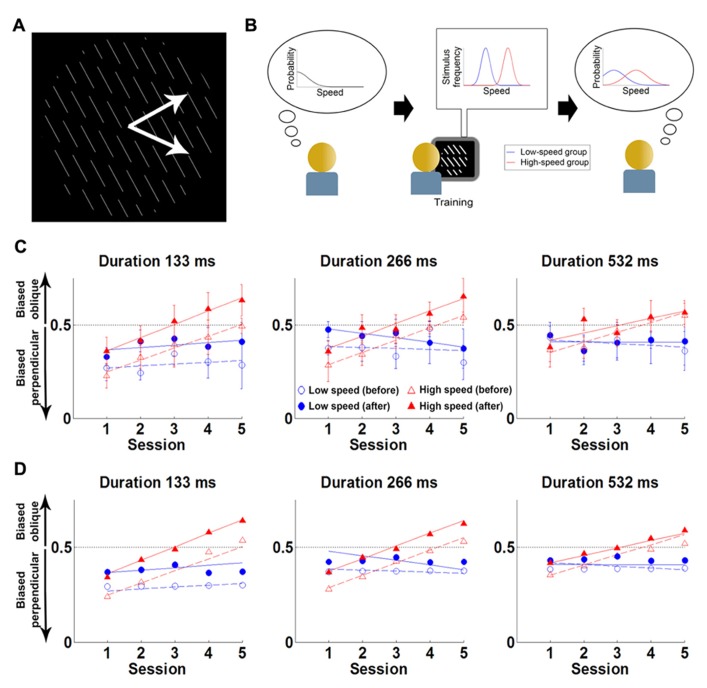
**Experiment and main results of Sotiropoulos et al. (2011a). (A)** The stimulus is a field of lines translating rigidly along either of the two directions shown by the white arrows (the latter are not part of the stimulus). The task of the participants is to report the direction of motion (“up” or “down”), without feedback. **(B)** Cartoon of experimental hypothesis. Left: initially participants have a prior favoring slow speeds. Middle: the low-speed group was exposed to low speeds (blue), while the high-speed group viewed faster speeds (red). Right: training will lead the high-speed group to shift their prior expectations toward higher speeds (red) compared to the low-speed group (blue). **(C)** Results: Proportion of oblique perceptions (*p*_o_) in low-contrast condition, for three trial durations. Each point is the *p*_o_ for the first (empty symbols) or last (filled symbols) test block of the session, for the high-speed (red) or the low-speed (blue) group. Lines correspond to linear fits to each block/group combination. Error bars denote between-subjects SEM. Initially participants are biased toward perceiving motion as being more often perpendicular to the orientation of the lines than it really is (consistent with estimating that the test stimulus is slower than it really is). However, this bias slowly decreases with training in the experimental group, and reverses after 3 days (consistent with estimating that the test stimulus is faster than it really is). **(D)** Fits from Bayesian model of motion perception (points) can account for the behavior of the two groups (lines, corresponding to the linear fits in **C**) when the speed prior is allowed to shift with training. Reproduced from Sotiropoulos et al., 2011a with permission.

They conducted a psychophysical experiment where participants were presented with a field of parallel lines translating rigidly along a direction that was either normal to the line (in 50% of the trials) or oblique to the line (in the other 50%). Participants were tested on their ability to report the perceived motion direction (normal or oblique) of the stimulus (**Figure 3**). The experiment was conducted over five sessions, taking place on consecutive days. Each session contained a short test block, a long “training” block and a final test block. The test blocks were always conducted with slow stimulus speeds (4°/s). The training block differed across groups: a control group performed the task at slow speeds (4°/s) and the experimental group at fast speeds (8°/s). The reasoning was that participants in the experimental group might implicitly update their expectations toward faster speeds, and thus experience a change in the direction bias.

Consistent with previous findings (Lorenceau et al., 1993), for low contrast stimuli, both groups initially perceived motion as being more often normal to the line than it really was. However, in the experimental group, this illusion gradually changed through the experience of the faster speeds, until the illusion reversed and the motion direction was perceived as being more often oblique than it really was. For the control group the illusion was unaltered.

The data was modeled using the Bayesian model of Weiss et al. (2002) described above. Sotiropoulos et al. (2011a) found that this model satisfactorily fit the data when extended to allow the speed prior to shift toward higher speeds with exposure. Overall, this suggests that the visual system expects a priori that the speeds of moving stimuli are slow but that the prior on slow speeds is not fixed and can change through implicit learning over the course of a few training sessions. After training, participants experienced clear perceptual biases about motion direction that were consistent with expecting that visual objects move quickly rather than slowly. Interestingly, expectations were found to update over two time-scales. First, within each session, participants exhibited a fast update of the prior (perhaps evidence of a contextual expectation). Second, this learning partially survived until the session of the following day. As a result, across sessions, a slow learning component was observed, with a modest shift of the prior from day to day (possibly evidence of a more “structural” expectation). These findings provide evidence for a causal link between the existence of the slow-speed prior and the learning of the statistics of stimuli in the world and show that structural priors can be updated throughout the lifetime.

## NEURAL SUBSTRATE OF EXPECTATIONS AND PRIOR BELIEFS

There is a well-known (and often criticized) gap between Bayesian model descriptions, which (by nature) account for cognitive processes at a computational level, and our understanding of the potential neurobiological mechanisms (see, e.g., Colombo and Seriès, 2012; Bowers and Davis, 2012; O’Reilly et al., 2012). Similarly, while there is some indication that priors and likelihood would be encoded separately in the brain (Beierholm et al., 2009; Vilares et al., 2012), not much is known about the neural substrate of expectations and priors. Whether (all) prior expectations correspond to top-down signals modulating early visual pathways, whether they reside entirely in higher-level areas, or on the contrary whether they are formed in sensory cortex itself is not clear. Moreover, prior expectations have been proposed to correspond to either a reduction of neural responses, or on the contrary to an increase in activity, a change in baseline activity, or a shift in the selectivity of the neurons activated by the expected features (Summerfield and Egner, 2009; Fiser et al., 2010; Vilares et al., 2012). We review some of the pertinent literature, which is limited, discuss some of the outstanding questions in the field (see Outstanding Questions) and suggest future directions that we believe will help resolve these issues (see Future Directions).

### PRIORS AND THE ACTIVITY LEVEL OF NEURONS SENSITIVE TO EXPECTED FEATURES

Unfortunately, there has been little investigation looking specifically at the effect of structural expectations at the level of individual neurons. There is, however, some indication that structural expectations could correspond to an enhancement of activity of the neurons sensitive to expected features. For example, structural expectations that objects are smooth in space or that lines would be co-circular (a.k.a. the Gestalt laws of proximity, continuity, co-circularity) have been linked with center/surround effects in V1 (for a review, Seriès et al., 2003). In situations of uncertainty (i.e., low contrast), such effects are mediated by neural response facilitation. For example the response to a low contrast bar is facilitated by the presence of a collinear surround.

A number of studies have also examined electrophysiological correlates of short-term learning of expectations. For example, Platt and Glimcher (1999) examined how decision variables in lateral intraparietal (LIP) area of awake monkeys are modulated by the frequency and reward associated with particular choices. They found that neural activity in LIP neurons was enhanced in neurons signaling the more likely response. Similarly, Basso and Wurtz (1997) investigated the influence of target uncertainty (defined by the number of stimuli from which a selection must be made) on the activity of superior colliculus neurons and found that activity preceding target selection increased when prior probability increased. However, whether such modulation would also be observed at earlier cortical stages (e.g., medio-temporal (MT) area) is not known.

Many electrophysiological studies have also manipulated contextual expectations so as to direct attention, using explicit external cues directed to particular locations or features. It is well-established in this case that directing attention to a location or feature in anticipation of a target leads to enhancement of activity in regions of the visual cortex that are selective for this location or feature (for a review, see, e.g., Carrasco, 2011).

By contrast, a number of fMRI and EEG studies have looked at the influence of stimulus repetition on neural activity, in a situation of passive viewing or using an “oddball” task. Those studies suggest that expectations correspond not to an enhancement of activity evoked by the expected stimuli but to a reduction (for a review, see Summerfield and Egner, 2009). Summerfield et al. (2008), for example, found that repetition suppression was reduced in the fusiform face area when repetitions of face images were improbable (and thus, unexpected). However, this result was not replicated in studies of non-human primates (Kaliukhovich and Vogels, 2011).

A possible reconciliation between fMRI studies finding a decrease in activity and single-unit studies finding an increase is that learning can result in a relative reduction of activity in neurons that are not selective to the expected feature or task compared to those that are selective (Adab and Vogels, 2011). Some data seems to be in line with this idea. For example, using an orientation discrimination task (with a contextual cue predicting the global orientation of the subsequent stimuli), De Lange and colleagues argue that (contextual) expectations, when behaviorally relevant, correspond not only to a decrease of activity but also a sharpening of the representation in visual cortex. They find that perceptual expectation leads to a reduction in neural activity in V1, but improves the stimulus representation, as measured by multivariate pattern analysis (Kok et al., 2012).

In line with this idea, there has been much attention to the selectivity of neurons involved in learning.

### PRIORS IN THE SELECTIVITY OF THE NEURONS

A natural way in which (structural) priors could be represented in the brain is in the selectivity of the neurons and the inhomogeneity of their preferred features (Ganguli and Simoncelli, 2010; Fischer and Pena, 2011; Girshick et al., 2011). In this framework, the neurons representing the expected features of the environment would be present in larger numbers (Girshick et al., 2011), or be more sharply tuned (Schoups et al., 2001), or more strongly connected to higher processing stages (Raiguel et al., 2006) than neurons representing non-expected features. For example, as discussed above, a Bayesian model with a prior on cardinal orientations (reflecting the fact that they are more frequent in the natural environment) can account for the observed perceptual bias toward cardinal orientations. These effects can also be simply accounted for in a model of the visual cortex where more neurons are sensitive to cardinal orientations, with those neurons being also more sharply tuned (as observed experimentally), combined with a simple population vector decoder (Girshick et al., 2011). Similar models have been proposed in the auditory domain to explain biases in localization of sources (Fischer and Pena, 2011) and formalized theoretically. Ganguli and Simoncelli (2010), for example, provided a thorough analysis of how priors could be implicitly encoded in the properties of a population of sensory neurons, so as to provide optimal allocation of neurons and spikes given some stimulus statistics. Interestingly, their theory makes quantitative predictions about the relationship between empirically measured stimulus priors, physiologically measured neural response properties (cell density, tuning widths, and firing rates), and psychophysically measured discrimination thresholds (see also: Wei and Stocker, 2012).

Whether all structural priors correspond to inhomogeneities in cell properties is unclear. The light-from-above prior is thought to be related to activity in early visual cortex (Mamassian et al., 2003), but, as far as we know, its precise relation with neural responses is yet unclear. The slow-speed prior, however, could be implemented in such a way, via an over-representation of very slow speeds in MT or a shift of the tuning curves toward lower speeds when contrast is decreased (Krekelberg et al., 2006; Seitz et al., 2008). Accordingly, there is some evidence that prolonged experience with high-speeds leads to a shift of the MT population to prefer higher speeds (Liu and Newsome, 2005).

### PRIORS IN THE NEURONS’ SPONTANEOUS ACTIVITY

Finally, an intriguing idea that has recently attracted much interest is that spontaneous activity in sensory cortex could be interpreted as samples of the prior distribution (Fiser et al., 2010; Berkes et al., 2011). The logic is the following. In a probabilistic framework, if neural responses represent samples from a distribution over external variables, this distribution is the so-called “posterior distribution.” By definition, the posterior distribution results from the combination of two components: the sensory input, and the prior distribution describing *a priori* beliefs about the sensory environment (i.e., expected sensory inputs). In the absence of sensory inputs, this distribution will collapse to the prior distribution, and spontaneous activity will correspond to this prior. This hypothesis would explain why spontaneous activity is found to be remarkably similar to evoked activity. Moreover, it would be computationally advantageous, driving the network closer to states that correspond to likely inputs, and thus shortening the reaction time of the system (Fiser et al., 2010). Berkes et al. (2011) recently provided further evidence for this idea by analyzing spontaneous activity in the primary visual cortex of awake ferrets at different stages of development. They found that the spontaneous activity is similar to the averaged evoked activity, with a similarity that increased with age and is specific for natural scenes. That spontaneous activity could correspond to the prior is a very attractive idea. More experimental and theoretical work is needed, however, to understand the validity, generality, and implications of this hypothesis. For example, whether spontaneous activity is mostly shaped by visual experience or by developmental programs is unclear. Similarly, it is yet unclear whether spontaneous activity could represent both structural and contextual expectations.

## OUTSTANDING QUESTIONS

Given that the learning of expectations has been addressed through diverse techniques and that many of the discussed studies were not explicitly designed to understand expectations, numerous questions remain at both the physiological and behavioral levels.

At the physiological level, a primary question is whether existing data about the neural effect of expectations can be unified in the same framework. For example, can we reconcile whether expectations lead to enhancement or suppression of neural activity (Summerfield and Egner, 2009)? Notably, single-unit recording studies and fMRI studies image different components of neuronal responses and different neuronal populations. For example, fMRI studies are influenced by changes across the all neurons in an area, perhaps favoring unselective neurons (as discussed above). Another possibility is that the imaging data may reflect mostly inhibitory activity, while extracellular recordings corresponds mostly to the activity of excitatory cells (Niessing et al., 2005; Gieselmann and Thiele, 2008). Expectations would then correspond to decreased inhibition. The effect of expectations might also depend on the behavioral relevance of expected stimuli: sensory signals that are behaviorally relevant would be enhanced, while expected stimuli that are irrelevant to the task at hand would be filtered out and suppressed (such as in repetition suppression). Another factor to consider is the time-scale of these effects. Chopin and Mamassian (2012) found that visual adaptation could lead to negative correlation of the current percept with visual events presented just before (<3 min) and a positive correlation with a remote reference window of stimuli (from 2 to 10 min in the past). They propose that the visual system uses statistics collected over the more remote past as a reference that is then combined with recent history for predicting the next percept. The most likely forthcoming percept would be the one that helps the statistic of the most recent percepts match that of the remote past. Perception would be biased toward such predictions when a new stimulus appears.

At the behavioral level, an issue that deserves further investigation concerns the dynamics of prior learning. In particular, how fast is learning compared to the optimal? Also, what information is stored from trial to trial about the prior distributions? A number of studies suggest that observers integrate information sub-optimally when learning stimulus statistics (e.g., Eckstein et al., 2004; Raviv et al., 2012). For example, using a 2-tones discrimination task, Raviv et al. (2012) show that participants exhibit (so-called “contraction”) biases that are consistent with using a prior that corresponds to the stimulus distribution. However, the most recent trials are found to be overweighted compared with the predictions of a standard Bayesian model, which can be interpreted as if the participants assume that the statistics of stimuli in the experiment is highly volatile. They suggest that Bayesian-like computation is approximated using a much simpler algorithm, in which the prior distribution is not fully represented. For example, their data can be accounted for by participants using only a single scalar to represent past trials, corresponding to an exponentially weighted sum of the current and past stimuli and their respective encoding noises.

Another crucial issue that needs to be clarified concerns the specificity of expectations. While specificity and transfer have been extensively studied in the context of perceptual learning (e.g., for a review: Seitz and Dinse, 2007; Sagi, 2011), only a few studies have investigated how specific prior expectations are (e.g., Adams et al., 2004; Adams, 2007; Maloney and Mamassian, 2009; Turk-Browne and Scholl, 2009; Gekas et al., 2013). For example, is there only one speed prior, which is applied to all types of visual objects and stimuli? When new priors are learned in the context of a task, do they automatically transfer to different tasks? When a structural prior seems to be over-ridden by short-term learning of the current statistics, is the initial representation maintained or over-written? Adams et al. (2004) provide evidence that the visual system uses the same prior about light source position in quite different tasks, one involving shape and another requiring lightness judgments. Similarly, Adams (2007) measured the “light-from-above” in different tasks: visual search, shape perception, and a novel reflectance-judgment task. They found strong positive correlations between the light priors measured using all three tasks, suggesting a single mechanism used for “quick and dirty” visual search behavior, shape perception, and reflectance judgments. In the context of short-term statistical learning, and using a familiarization task with complex shapes, Turk-Browne and Scholl (2009) provide evidence for transfer of perceptual learning across space and time, suggesting that statistical learning leads to flexible representations. Similarly, the findings of Gekas et al. (2013) suggest that human observers are prone to transfer between similar stimulus configurations. However, the generality of these findings is very unclear and needs further exploration. A related question is to understand how long expectations learned in the laboratory persist over time. This question and that of transfer are in fact crucial for assessing whether our classification into “contextual” and “structural” priors is meaningful. If contextual priors can persist for long periods (Olson and Chun, 2001; Kim et al., 2009; Adams et al., 2010; Sotiropoulos et al., 2011a), transfer to different tasks (Adams et al., 2004; Turk-Browne and Scholl, 2009) and more importantly to different contexts, it would suggest that the mechanisms that lead to contextual priors are similar to those which lead to the formation of structural priors (Fiser and Aslin, 2001, 2002). Contextual expectations could become structural over time. Kerrigan and Adams (2013) suggest, however, that contextual priors persist over time, but remain context-dependent [although possibly not stimulus-dependent (Adams et al., 2004)], with the experimental set-up acting as a contextual cue. More evidence is needed so as to test the generality of this finding.

This issue of flexible representations also brings to question the extent to which learning of expectations and classical perceptual learning rely upon similar mechanisms. Perceptual learning is commonly defined as changes in perceptual processing that occur with experience (typically through active training on a given task) and is a phenomenon that influences nearly all aspects of vision. Examples of perceptual learning range from the abstract, such as pattern recognition found in expert chess players, radiologists, and visual aspects of language processing, to intermediate levels of processing such as those found in categorical, associative, and object learning, to low-level perceptual learning of basic visual skills such as contrast detection, orientation discrimination, and hyperacuity judgments, etc. While these different visual processes may not be perfectly dissociable, it is clear that perceptual learning is used to describe aspects of learning that involve a myriad of visual processes, that result from plasticity in a diverse set of brain areas and certainly includes phenomena that we have discussed regarding both contextual and structural expectations. There is already great debate about the different mechanisms in the brain that subserve perceptual learning; such as reducing the system’s noise (Dosher and Lu, 1998), increasing the gain of the signal (Gold et al., 1999), improving an internal template of the target (Li et al., 2004), better attending the location or features of the stimulus (Franko et al., 2010), improving decisions rules regarding the stimulus (Zhang et al., 2010), among other mechanisms. It would be very valuable to assess how these mechanisms and related debates, could apply, or not, to expectation learning. It may be, for example, that structural and contextual priors differ in how they are learned and at which stage of processing. Similar to the perceptual learning literature (e.g., for a review, Sagi, 2011; Sotiropoulos et al., 2011b; Choi and Watanabe, 2012), one might wonder whether structural expectations could be understood in terms of a change in representation in perceptual areas, while contextual expectations could correspond more to top-down signals coming from decision stages and resulting in a selection (or “reweighting”) of the sensory signals. Similarly, in Bayesian terms, we have focused on how expectations and learning could be described by changes in sensory priors, but these are only one element of the internal model thought to be used by the brain. In theory, perceptual learning could correspond to changes at different levels: changes in prior distributions, but also changes in sensory representations (perceptual likelihood), and changes in decision rules or read-out strategies. More work is needed to understand how perceptual learning maps onto changes of these different elements.

A related question regards the distinction between attention and expectations. These effects can easily be confounded, in experimental designs and results interpretation. In many situations, stimulus expectations can direct attention to particular visual features and locations (Posner, 1980; Downing, 1988). In addition, attention and expectations are generally thought to be controlled by similar cognitive processes, which allocate increased resources to the perceptual processing of stimuli that are either behaviorally relevant or contextually likely (Corbetta and Shulman, 2002). Chalk et al. (2013) propose that what is usually described as “expectations” and “attention” might correspond to two sides of the same mechanism. They propose that the visual system is constantly seeking to optimize its internal model so as to predict how the sensory input and reward received for performing different actions are generated by a common set of hidden causes (Sahani, 2004). In this Bayesian model, goal-oriented attention and expectations refer to adaptation of the system’s priors to changes in reward statistics (or task relevance) and stimulus statistics, respectively. The model is consistent with, and provides a normative explanation for, recent divisive normalization models of attention (Reynolds and Heeger, 2009) and provides new tools for understanding how the brain’s internal models should change with task demands and stimuli statistics, but remains tractable unfortunately only in extremely simplified situations.

These outstanding issues (see also **Box 1**) suggest that there is much work to be done to better understand how expectations are learned and their behavioral manifestations.

**Box 1. Outstanding questions.**

(1) What are the limits in the complexity of expectations and prior distributions that can be formed?
(2) Are priors specific to the learned conditions? How do they transfer to similar stimuli, tasks, and/or contexts?
(3) How optimal is the learning of priors? Are there biological constraints limiting what can be learned and how fast? What heuristics would form plausible alternatives to Bayesian inference?
(4) Where and how are priors and likelihoods integrated?
(5) Are priors mostly encoded in the preparatory activity prior to the stimulus presentation, the modulation of evoked activity, or the read-out?
(6) How can we better disentangle expectations vs. sensory adaptation, perceptual learning, attention, working memory?

## FUTURE DIRECTIONS

We suggest that targeted studies need to be conducted to better understand the neuronal basis of structural expectations. Physiological experiments using statistical learning designs comparable with the behavioral studies are needed to clarify the neural basis of expectations, and whether contextual and structural expectations share the same mechanisms.

It would be very valuable, for example, to obtain electrophysiological recordings from brain regions such as MT and LIP in awake monkeys while performing tasks such as that of Chalk et al. (2010) and Sotiropoulos et al. (2011a) and measure change in responses properties with learning. This would help disentangle between different hypotheses. For example, the attractive biases observed in Chalk et al. (2010) are theoretically compatible with a model that assumes either an increase of activity for expected directions or a shift of the tuning curves toward the expected directions in MT combined with a read-out mechanism (e.g., in LIP) that does not change on a short time-scale (Seriès et al., 2009). The hallucination data could be explained by an increase in baseline activity for the expected directions. Alternatively, the data may be explained by reweighting mechanisms between the MT stage and the LIP decision stage (Law and Gold, 2008) that would favor the influence of neurons selective to the expected directions.

Another research question that needs further investigation is the link between Bayesian models and the biological substrate. Bayesian models of perception have been increasingly popular in the past 10 years. However, unfortunately, Bayesian models are usually aimed at describing performance and are not predictive at the neural level (Colombo and Seriès, 2012; Bowers and Davis, 2012; O’Reilly et al., 2012). One reason is that it is not known how (or whether) probability distributions are encoded by neurons. Moreover, there is a lack of computational models describing a neural implementation of probabilistic learning that would provide experimentally testable predictions (Fiser et al., 2010). To progress with these issues, further experiments and models will be needed. At the experimental level, investigating the time-scale and specificity of priors, as well as the limits of the complexity of the priors that can be learned will shed light into the biological constraints. We expect that priors formed over different time-scales will likely involve different forms of plasticity. At the theoretical level, we believe that it is crucial to propose plausible neural implementations of generative models of the sensory inputs (Lee and Mumford, 2003). To capture how expectations and internal priors are shaped by experience, such models would need to be able to learn neural representations from sensory data. To explain how priors influence ongoing perception, the models would need to connect perceptual processes to (approximate) probabilistic inference.

Furthermore, the utility of modeling approaches from other fields, such as those of machine learning, should be investigated in their potential to create better biological models. For example, Reichert et al. (2010, 2011, 2013) have recently proposed that the deep Boltzmann machine (DBM) offered a promising (even if idealized) description of a generative model that learns to synthesize representations of sensory data. A DBM can be seen as an instance of a hierarchical probabilistic model, and captures the intuition of bottom-up and top-down processing in the cortex reflecting the interaction between sensory information and internal priors. At the same time, the DBM is also a simple neural network, with “deep” organization into hierarchical layers and image-based representations that can be directly linked to properties of the visual cortex. Modeling neural mechanisms such as homeostatic plasticity in the generative framework, Reichert et al. (2010) show how an imbalance of bottom-up and top-down processing then can be seen as a cause for hallucinations to emerge, such as in Charles Bonnet syndrome. Reichert et al. (2011) further show how such a model can account for bistable perception as originating from sampling-based approximate probabilistic inference. Still, models such as the DBM leave much to be desired both in terms of biological realism and in terms of their capability to deal with realistic complex sensory input. Such challenges will need to be addressed by future work.

## CONCLUSION

Here, we introduced a conceptual framework by which to consider different types of expectations (contextual vs. structural; **Figure 1**). We reviewed how expectations can be studied using Bayesian models and described as probabilistic priors. Within this framework, we showed that such priors provide a parsimonious way to understand many perceptual phenomena, and that such priors can be measured in individuals. They are then found to approximate the stimulus statistics of the environment, with some individual variability that can be related to performance variability. Furthermore, new priors can be acquired throughout the lifetime, and existing priors appear to be shaped through our on-going experience in the world. While there exists a rich literature providing theoretical ideas and behavioral and neurophysiological data related to priors and expectations, the field has lacked a clear unifying framework. As such, many questions remain regarding both phenomenology and mechanisms. We suggest that investigating the neurobiological underpinnings of expectations might be a promising starting point for understanding how (approximate) Bayesian inference is implemented in the brain. We propose a few guidelines for further studies so as to bridge the gap between theoretical models, physiological and behavioral data.

## Conflict of Interest Statement

The authors declare that the research was conducted in the absence of any commercial or financial relationships that could be construed as a potential conflict of interest.
